# Pancreatic cyst development: insights from von Hippel-Lindau disease

**DOI:** 10.1186/2046-2530-2-3

**Published:** 2013-02-05

**Authors:** Sophie J van Asselt, Elisabeth GE de Vries, Hendrik M van Dullemen, Adrienne H Brouwers, Annemiek ME Walenkamp, Rachel H Giles, Thera P Links

**Affiliations:** 1 Department of Endocrinology, University of Groningen, University Medical Center Groningen, PO Box 30.001, 9700 RB, Groningen, The Netherlands; 2 Department of Medical Oncology, University of Groningen, University Medical Center Groningen, PO Box 30.001, 9700 RB, Groningen, The Netherlands; 3 Department of Gastroenterology, University of Groningen, University Medical Center Groningen, PO Box 30.001, 9700 RB, Groningen, The Netherlands; 4 Department of Nuclear Medicine and Molecular Imaging, University of Groningen, University Medical Center Groningen, PO Box 30.001, 9700 RB, Groningen, The Netherlands; 5 Department of Nephrology and Hypertension, University Medical Center Utrecht, Utrecht, The Netherlands

**Keywords:** Cilia, Cytoskeleton, Pancreatic cysts or serous cystadenomas, VHL tumor suppressor protein, von Hippel-Lindau disease

## Abstract

Pancreatic cysts are a heterogeneous group of lesions, which can be benign or malignant. Due to improved imaging techniques, physicians are more often confronted with pancreatic cysts. Little is known about the origin of pancreatic cysts in general. Von Hippel-Lindau (VHL) disease is an atypical ciliopathy and inherited tumor syndrome, caused by a mutation in the *VHL* tumor suppressor gene encoding the VHL protein (pVHL). VHL patients are prone to develop cysts and neuroendocrine tumors in the pancreas in addition to several other benign and malignant neoplasms. Remarkably, pancreatic cysts occur in approximately 70% of VHL patients, making it the only hereditary tumor syndrome with such a discernible expression of pancreatic cysts. Cellular loss of pVHL due to biallelic mutation can model pancreatic cystogenesis in other organisms, suggesting a causal relationship. Here, we give a comprehensive overview of various pVHL functions, focusing on those that can potentially explain pancreatic cyst development in VHL disease. Based on preclinical studies, cilia loss in ductal cells is probably an important early event in pancreatic cyst development.

## Review

### Introduction

Pancreatic cysts are frequent, with a prevalence of 2.4 to 13.5% in patients without known pancreatic disease. Due to increased use of cross-sectional imaging techniques, physicians are more frequently confronted with pancreatic cysts [1]. Various types of pancreatic cysts can occur, which can be benign or have malignant potential. An expectative policy is accepted for benign cysts and surgery is indicated for malignant lesions. Currently, accurate diagnostics are not available to identify malignant cysts [1]. Despite the need for mechanistic insight, little is known about the origin and pathophysiology of pancreatic cysts in general.

**Figure 1:**
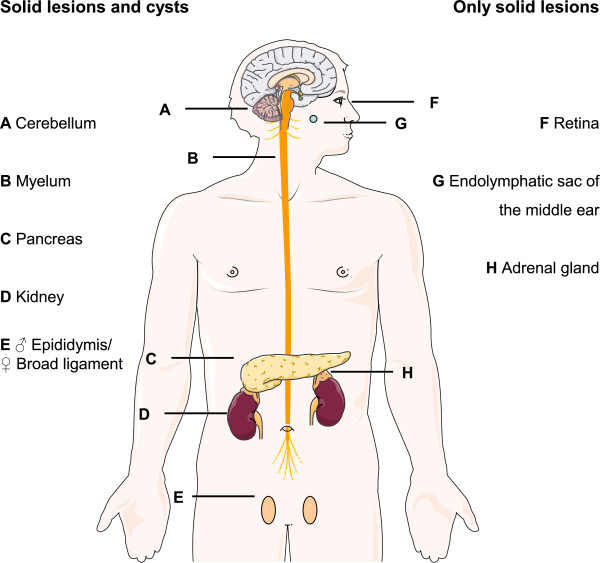
**VHL disease can affect various organs.** On the left, the organs in which cysts as well as solid lesions occur, have been listed and on the right are the locations in which only hypervascular solid lesions occur. (Constructed using Servier Medical art).

Von Hippel-Lindau (VHL) disease (MIM #193300) is a rare hereditary tumor syndrome that results from a germline mutation in the *VHL* gene. The reported incidence is 1 per 36,000 live births and a >90% penetrance is present by the age of 65 years [2]. VHL disease can lead to the development of hemangioblastomas of the central nervous system, retinal angiomas, endolymphatic sac tumors, epididymis or broad ligament cystadenomas, renal cysts and renal cell carcinomas (RCCs), pheochromocytomas, pancreatic cysts and pancreatic neuroendocrine tumors (pNETs) [3] (Figure 1). Currently, RCC and hemangioblastomas are the main causes of death [4,5]. VHL patients undergo screening for early detection of manifestations [6]. Understanding the role of the *VHL* gene in the oxygen-sensing pathway in the tumor micro-environment of RCC has led to major pharmaceutical successes through targeted therapies for many cancer types, such as humanized antibodies targeting vascular endothelial growth factor (VEGF), mTOR- and VEGF receptor tyrosine kinase inhibitors [7]. As a result, first-line treatment of metastasized RCCs has entirely changed in the last decade.

pNETs are present in 10 to 17% of VHL patients [8,9] and pancreatic cysts occur in about 70% [10,11]. Because of this high prevalence, it is worthwhile examining the early cellular events that result in pancreatic cysts in VHL disease, reflecting insight into pancreatic cystic disorders in general. In this review, we conduct a complete overview of pVHL functions to explain cellular events involved in cyst development in the context of VHL. Based on knock-out mouse models, we discuss the consequences of *Vhlh* loss in the pancreas and the origin of pancreatic cysts.

### Pancreatic involvement in VHL disease

**Figure 2:**
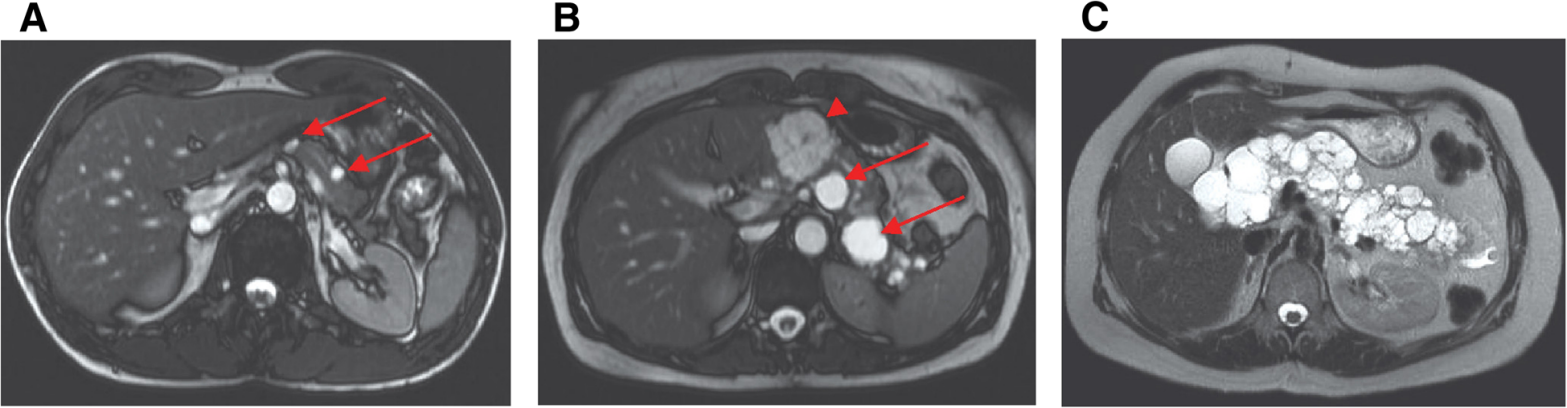
**Axial MRI images of pancreatic involvement in three VHL patients.** None of these patients had pancreas-related symptoms or exocrine/endocrine insufficiency. (**A**) Simple cysts (arrows) with size <1 cm in a 32 year old man; (**B**) A 4 cm sized serous microcystic cystadenoma (arrowhead) is present next to multiple simple cysts (arrows) in a 39 year old woman; (**C**) Shows replacement of almost the entire pancreas by multiple cysts in a 47 year old woman.

VHL pancreatic cysts include simple cysts and serous cystadenomas. In addition to these cysts pNETs occur in VHL patients, which can have malignant potential [10]. One autopsy series of 50 VHL patients showed a prevalence of 72% for pancreatic cysts [11]. In the largest clinical study describing pancreatic involvement, 158 VHL patients underwent abdominal computed tomography scan at least once. Pancreatic involvement was observed in 77% of patients: 71% had simple cysts, 15% had serous cystadenomas and 10% had pNETs, which coincided with pancreatic cysts in 11 cases (69%) [10]. In VHL patients, a broad heterogeneity is present regarding pancreatic cyst involvement: isolated cystadenomas and small cysts occur, whereas in some patients cystic growth replaces almost the entire pancreas (Figure 2) [10,12-16].

Data is limited about clinical consequences of VHL pancreatic cysts. One study [10] and numerous case-reports [12,13,17-23] have recorded clinical problems, of which compression of the biliary tract was most frequently reported (Table 1). Intervention was indicated in only 3% of VHL patients [10]. No evidence exists for an association between endocrine or exocrine pancreatic insufficiency and cyst involvement. Moreover, no cases have been described of malignant pancreatic cysts in VHL disease. Nineteen cases with a pancreatic serous cystadenoma mixed with a pNET were reported [10,14-16,24,25], but no relationship exists between presence of pancreatic cysts and pNETs. Conclusively, pancreatic cysts in VHL disease are not associated with malignancy and sporadically cause problems [26].

**Table 1:** Complications caused by pancreatic cysts in VHL disease

**Reference [No.]**	**No. of patients**	**Symptoms**	**Diagnosis**	**Therapeutic intervention**
[17]	1	Jaundice	Bile duct obstruction	Surgery
[19]	1	Jaundice	Bile duct obstruction	Biliary stent
[20]	2	Jaundice	Bile duct obstruction	Surgery
[12]	2	Jaundice	Bile duct obstruction	Surgery
[22]	1	Abdominal pain, jaundice and fever	Bile duct obstruction	Surgery
[23]	1	Pruritis	Bile duct obstruction	Surgery
[10]	2	Abdominal pain	Pressure/ obstruction	1 Drainage
				1 Surgery
[10]	1	Not reported	Necrotizing pancreatitis	Expectative
[21]	1	Vomiting	Pressure on stomach and duodenum	Surgery
[13]	1	Abdominal swelling	*Hemosuccus pancreaticus*	Surgery
[18]	1	Abdominal swelling	Duodenal compression	Surgery

### VHL disease classification

The clearest genotype-phenotype correlation is exemplified by type 2 VHL, typically characterized by a *VHL* missense mutation and presence of pheochromocytomas [27,28]. Type 1 VHL is more frequently characterized by a *VHL* truncating mutation and absence or rare occurrence of pheochromocytomas. Type 2 *VHL* alleles can be further subdivided based on absence or presence of RCC; called VHL type 2A and type 2B, respectively [28,29]. A pheochromocytoma-only subtype has also been described: VHL type 2C [30]. Pancreatic involvement occurs in both VHL type 1 and type 2B, although it is unclear whether it occurs in the rare VHL types 2A and 2C [31].

### The *VHL* gene

The *VHL* gene was identified in 1993 [32] and is a tumor suppressor gene; somatic inactivation of the wild-type allele or loss of heterozygosity (LOH) of the *VHL* gene is often observed prior to development of VHL-associated lesions [33,34]. Consisting of three exons, the human *VHL* gene is located on chromosome 3 (3p26-p25), encoding a 213-amino acid pVHL (30 kDa VHLp30) and a 160-amino acid shorter form (19 kDa VHLp19) [35,36]. The role of *VHL* in the oxygen-sensing pathway is its best-characterized function: cellular normoxic conditions enable the pVHL E3 ubiquitin ligase complex to target the α-subunit of hypoxia inducible factor (HIF) for proteosomal degradation. During hypoxia, pVHL is not able to bind HIF-α, resulting in accumulation of un-ubiquitinated HIF-α, which then translocates to the nucleus. This stimulates the transcription of various genes, including *VEGF*[37].

Since pVHL is still capable of functioning within the E3 ubiquitin ligase complex in VHL type 2C [38], other pVHL functions must be present to induce tumorigenesis. Indeed, *VHL* also regulates the assembly of the extracellular matrix (ECM) [39-45], and recent studies have shown that pVHL regulates the microtubule cytoskeleton, particularly plus-end stability [46-54].

### Sporadic pancreatic serous cystadenomas

Data suggest that *VHL* loss through LOH of chromosome 3p might be a common mechanism initiating cystogenesis in sporadic pancreatic cystadenomas [55,56]. Recently, whole-exome sequencing was performed in various sporadic pancreatic cysts [57]. Seven out of eight serous cystadenomas lost chromosome 3p alleles, and in four, *VHL* gene mutations were found. This further supports that *VHL* loss initiates cystogenesis in pancreatic cystadenomas. Given that loss of the *VHL* locus was the only recurrent lesion identified, these data indicate that *VHL* loss alone could be sufficient for this development. Interestingly, in this same study, intraductal papillary mucinous neoplasms, mucinous cystic neoplasms, and solid pseudopapillary neoplasms did not show alterations in 3p alleles. In these lesions other genes encoding E3 ubiquitin ligases were involved, pointing to protein turnover as an underlying mechanistic theme [57].

### pVHL regulates cellular architecture

**Figure 3:**
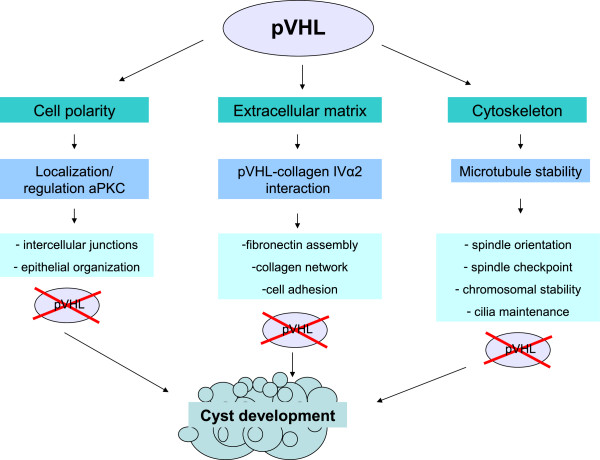
Schematic overview of functions of pVHL.

Most *in vitro* studies exploring the influence of pVHL on the cytoskeleton have been performed in RCC cell lines. To the best of our knowledge, no comparable studies have been performed with pancreatic cell lines. Since somatic LOH of wild-type *VHL* allele has been verified in pancreatic cysts in VHL [58], we reviewed the existing literature based on RCC cell line studies to gain insight into the effect of pVHL on pancreatic cell regulation and cyst development. Figure 3 represents a schematic overview of pVHL functions which might also explain pancreatic cyst development in VHL disease.

### pVHL and the extracellular matrix

**Figure 4:**
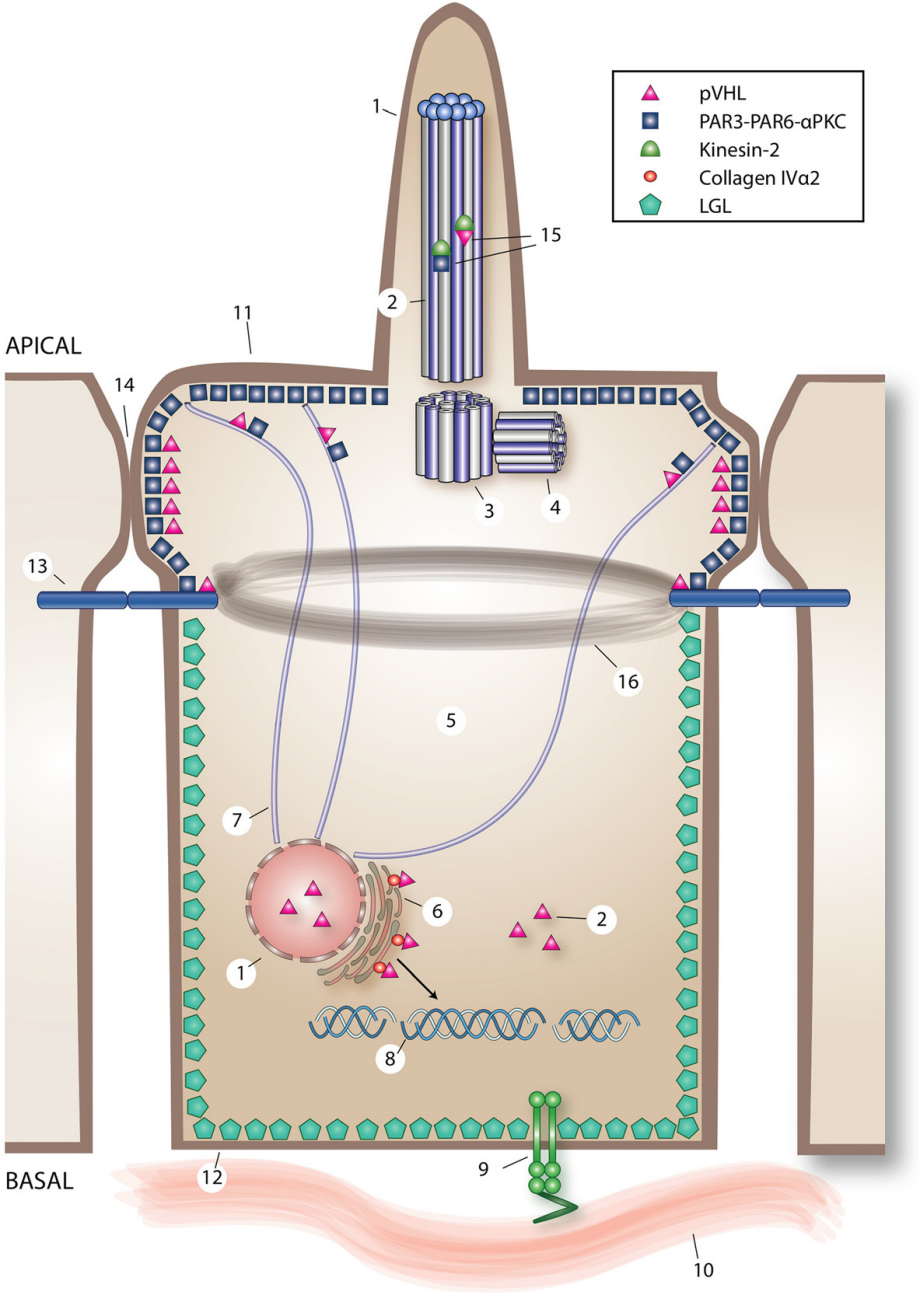
**Overview of direct and indirect pVHL-regulated cell processes in an epithelial cell.** The cilium (1) consists a microtubule-based axoneme (2), and a mother (3) and a daughter centriole (4). In the cell, pVHL is located in the cilium (1), the cytoplasm (5), the endoplasmic reticulum (6) and microtubules (7). The endoplasmic reticulum is the cellular compartment where trihelical collagen IV (8) is produced. pVHL binds collagen IVα2 in the endoplasmic reticulum. When pVHL-collagen IVα2 binding is perturbed, defects in the collagen network results. Integrins (9) facilitate cell-ECM (10) adhesion. Upon loss of pVHL function, misregulation of β1-integrin disturbs the fibronectin matrix assembly. The epithelial cell polarity complex PAR3-PAR6-aPKC is located at the apical membrane (11) and LGL2 at the basolateral membrane (12). Moreover, pVHL and PAR3-PAR6-aPKC are necessary for the formation of adherens junctions (13) and tight junctions (14). PAR3-PAR6-aPKC and pVHL play a role in regulation of the cilium, each capable of binding kinesin-2 (15). Functional loss of pVHL destabilizes cell polarity, partially attributable to abnormal adherens junctions [with consequent effects on the actin belt (16)] and unstable tight junctions. The relevant literature supports a scenario whereby microtubule instability as a result of pVHL dysfunction might affect PAR3-PAR6-aPKC localization, subsequently destabilizing cell polarity and cilia maintenance.

The ECM consists of proteins including collagen, fibronectin and laminin. Fibronectin plays a major role in the spread and migration of cells by binding them to the ECM. Integrins are cell surface receptors that mediate cell-cell and cell-ECM attachment [59]. pVHL promotes cell adhesion to the ECM [40]. *VHL* inactivation in RCC cells, mouse embryos and mouse embryo fibroblasts impair the ability to form a fibronectin assembly [39]. Fibrillar adhesions are essential to form a fibronectin assembly. Despite sufficient fibronectin, *VHL−/−* RCC cells fail to construct β1-integrin fibrillar adhesions due to deficient integrin regulation [41]. In RCC cell lines with wild-type *VHL,* collagen IV interacts with pVHL [42]. More specifically, pVHL binds the collagen IVα2 chain, part of the triple helix collagen IV; whereas in RCC cells with mutant pVHL this interaction fails [43]. This results in loss of collagen network *in vitro* and collagen remodeling *in vivo* (Figure 4) [42]. Collagen IV associates with fibronectin, suggesting that the previously observed interaction between pVHL and fibronectin is indirect [43].

In a xenograft model using RCC cell lines, VHL-ECM, VHL-HIF or both pathways were inactivated. *VHL−/−* 786–0 cells expressing HIF-2α failed to assemble an ECM. *VHL+/+* cells retrovirally infected to produce proteasome-resistant HIF-2α, were still able to assemble an ECM, indicating that it is independent of VHL-HIF regulation. Alternatively in VHL type 2C cells, mutant pVHL regulates HIF normally, but interferes with ECM assembly [45]. Xenograft tumors from VHL type 2C cells were hypervascular and invasive, similar to tumors originating from *VHL−/−* 786–0 cells. In contrast, xenografts derived from *VHL+/+* cells engineered to stabilize HIF-2α resulted in tumors with lower microvessel density and invasiveness, despite higher VEGF expression [45]. Thus, upregulation of VEGF alone is not sufficient for hypervascularization of tumors. Kurban *et al.* suggested that a strong collagen IV network, dependent on pVHL, naturally suppresses tumorigenesis [45].

### pVHL and cell polarity

Epithelial cells have asymmetric specification of membrane domains. Asymmetry and polarization are regulated by partitioning defective proteins (PAR) and atypical protein kinase C (aPKC). The PAR3-PAR6-aPKC complex is essential for establishing the apical membrane domain and junction structures of epithelial cells [60]. In addition, this complex is involved in formation of the apical lumen in three-dimensional cultures [61].

The pVHL ubiquitin ligase complex targets the active form of aPKC for degradation, analogous to HIF-α [62]. *VHL* mutant cells fail to form intercellular junctions, resulting in lost polarity [44]. This may be due to deregulation of active aPKC. Moreover, pVHL associates with the PAR3-PAR6-aPKC complex [50]. In a follicular epithelial model in *Drosophila,* loss of wild-type *vhl* resulted in epithelial disorganization, microtubule destabilization and subsequent aPKC mislocalization [63] (Figure 4). Duchi *et al.* concluded that loss of *VHL in vivo* can destabilize strict planar cell polarity control, resulting in architectural changes permissive to cyst development [63].

### pVHL and microtubule dynamics

Microtubules are polymerized filaments composed of α- and β-tubulin monomers. Microtubules continuously shrink at their “minus-ends” and grow at their “plus-ends” [64]. pVHL promotes microtubule stabilization by reducing tubulin turnover [46,47,54]; it binds microtubules through kinesin-2 [48]. *In vitro* inhibition of tubulin GTP-ase activity by pVHL at microtubule plus-ends contributes to this stability, which is compromised by *VHL* patient-associated mutant alleles [54]. Furthermore, cellular inactivation of pVHL results in spindle misorientation, spindle checkpoint weakening and chromosomal instability attributed to microtubule instability [53]. Interestingly, pVHL directs growth of microtubule orientation towards the outer plasma membrane [50].

### pVHL and cilia

Microtubules form the backbone of cilia, which project from the apical cell surface. Cilia sense outside the cell and are involved in signaling pathways. Intraflagellar transport of ciliary components is required for ciliary functions, which is powered by kinesin-2. The heterotrimeric motor kinesin-2 comprises motor subunits of kinesin superfamily protein 3 (KIF3A, KIF3B) and kinesin-associated protein 3 (KAP3) [65]. pVHL binds KIF3A and KAP3 of kinesin-2 [48], and in the cell, mobility of pVHL is at least partially regulated by kinesin-2 [49].

In renal tissue from VHL patients, cilia are lost in cysts, while cilia are still present in normal tissue [51]. Accordingly, RCC cell lines with or without reconstitution of wild-type *VHL*, show that pVHL contributes to ciliary maintenance and stability [51,52]. Cilia loss in kidney tubules due to pVHL dysfunction likely results from disoriented microtubule growth and decreased microtubule stability [50], and is associated with renal cyst development (Figure 4) [51]. It has not been confirmed that pancreatic cysts in VHL patients are the consequence of cilia loss. However, in a pancreatic-specific *Kif3a* knock-out mouse model, cyst development was attributed to cilia loss (see “Cilia loss in pancreatic cells *in vivo*”) [66]. Therefore, it is likely that pancreatic cysts in VHL are a result of similar consequences.

### Histopathology of the pancreas in VHL

**Figure 5:**
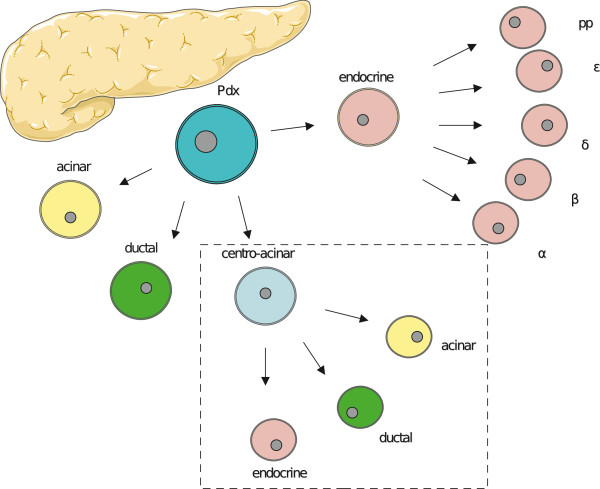
**Pancreatic progenitor or Pdx-cells can differentiate into endocrine or exocrine cells, including acinar, ductal and centro-acinar cells.** Strong evidence supports the assertion that centro-acinar cells, similar to Pdx-cells, can differentiate into both endocrine and exocrine cells (Constructed using Servier Medical art).

Embryonic epithelial cells that express the transcription factor pancreatic and duodenal homeobox 1 (Pdx1) give rise to pancreatic tissue, consisting of exocrine (acinar and duct) and endocrine (islet) cells [67]. Centro-acinar cells are duct cells, which connect acini with intralobular ducts. Recently centro-acinar cells were isolated based on specific expression of aldehyde dehydrogenase 1. *In vitro*, centro-acinar cell suspensions were able to proliferate with a capacity to differentiate into both exocrine and endocrine cells [68]. Centro-acinar cells might therefore act as facultative progenitor cells in the mature pancreas for acinar, duct and islet cells (Figure 5).

### Histology of pancreatic cysts and pNETs

Pancreatic cysts and pNETs in VHL disease have distinct features. pNETs have a solid, trabecular and/or glandular architecture with stromal collagen bands and neurosecretory dense core granules [69], which are absent in VHL pancreatic cysts [58]. Immunohistochemical staining for chromogranin A, S-100, synaptophysin and neuron-specific enolase showed positive expression in VHL pNETs [69]. Pancreatic cysts stained negative for chromogranin A and S-100 [58]. The histology of 119 pancreatic cysts was examined in detail from nine VHL patients [58]. All demonstrated a mixture of clear and/or amphophilic glycogen-rich epithelial cells, endothelial cells and smooth muscle cells. Cysts contained and were surrounded by fibrous tissue. In both pNETs and pancreatic cysts, LOH of *VHL* wild-type allele was confirmed [58,69].

### The pancreas in VHL mouse models

Constitutional inactivation of *Vhlh* results in embryonic lethality at 10.5 to 12.5 days of gestation, due to placental vasculogenesis defects [70]. To investigate the development of VHL-associated pancreatic manifestations, conditional mouse models have been generated using *Cre/LoxP* technology [71]. In other mouse models *Vhlh, Hif-1α* or both, were conditionally inactivated in pancreatic β-cells in order to investigate the role of pVHL in glucose metabolism [72-75].

In the first study [71], the *Vhlh* gene was inactivated in pancreatic progenitor cells by driving *Cre* recombinase with the *Pdx-1* promoter. Postnatal death was observed in the *Pdx1-Cre;Vhlh f/f* mice (n = 22), of which 18 were dead within five days. Histological analyses by pathologists blinded for genotypes showed no abnormalities. However, five individual *Pdx1-Cre;Vhlh f/f* mice survived longer. At 6 to 7 months of age, no pancreatic abnormalities were found in two *Pdx1-Cre;Vhlh f/f* mice. The remaining three were sacrificed at 16 to 18 months of age. At this time point, pancreatic tissue was replaced by fat deposition and pancreatic cysts, and microcystic adenomas were present. The epithelial lining and endothelial cells of the microcystic adenomas expressed cytokeratin MAK6 and CD31, respectively, comparable to findings of pancreatic cysts in VHL patients [58]. In *Pdx1-Cre;Vhlh f/f* mice, all pancreatic islets were characterized by complex and dilated hypervascularity. Some islets had a small, abnormally shaped appearance and others were hyperplastic [71]. In another study *Pdx1-Cre;Vhlh f/f* mice were born in the normal Mendelian frequencies. At 12 months of age no cysts or tumors were seen, but a slightly increased pancreatic vascularization was present, compared to control mice [73].

In other knock-out mouse models the *Vhlh* gene was inactivated by targeting endocrine pancreatic cells for *Cre* recombinase [71]. In mice with conditional *Vhlh* inactivation of endocrine α-cells or β-cells, no pancreatic abnormalities were observed (n = 16). Deletion of *Vhlh* alleles in α- or β-cells was confirmed by polymerase chain reaction analysis [71]. However, others did find increased vascularization in islets in conditional *Vhlh* knock-out mice in β-cells [73-75]. Additionally, disrupted islet morphology with α-cells scattered throughout the islets were found in *Vhlh* knock-out mice in β-cells [75]. In all these models, no pNETs were observed. Table 2 shows an overview of knock-out mouse models, serving as a VHL pancreatic model.

**Table 2:** VHL (related) pancreatic knock-out mouse models

**Reference [No.]**	**Knock-out gene(s)**	**Target cells**	**Age (months)**	**Pancreatic manifestations**
[71]	*Vhlh*	Progenitor	6-8	None
[73]	*Vhlh*	Progenitor	12	Increased pancreatic vascularization
[71]	*Vhlh*	Progenitor	16-18	Cysts, microcystic adenomas, fat depositions, abnormal shaped and hyper-vascular islet cells
[71]	*Vhlh*	Endocrine α	10-23	None
[71]	*Vhlh*	Endocrine β	15	None
[73]	*Vhlh*	Endocrine β	12	Increased vascularization in islets
[72]	*Vhlh*	Endocrine β	6.5	None
[72]	*Vhlh; Hif-1α*	Endocrine β	6.5	None
[72]	*Hif-1α*	Endocrine β	6.5	None
[74]	*Vhlh*	Endocrine β	N/A	Increased vascularization in islets
[75]	*Vhlh*	Endocrine β	2	Increased vascularization and α-cells scattered throughout the islets
[66]	*Kif3a*	Progenitor	2	Compromised acinar tissue
[66]	*Kif3a*	Progenitor	12	Fibrosis, ductal dilation, cysts

### Origin of pancreatic lesions in VHL

Whether pNETs originate from exocrine or endocrine cells remains unknown [76]. In pancreatic tissue of 13 VHL patients who underwent surgery because of pNETs, microadenomas were found ranging from 1 to 25 per patient. Expression of cyclin D1, carbonic anhydrase 9 and HIF-1α suggests that these microadenomas occur due to LOH of *VHL* in clonal lesions, which might be an early stage of pNETs. Most microadenomas were located close to acinar cells, but were also found close to duct or islet cells [76].

In contrast, evidence exists that pancreatic serous cystadenomas originate from duct cells. In these cysts and duct cells, co-expression of cytokeratin patterns is present, as determined immunohistochemically [77,78]. Moreover, of 38 serous cysts including two VHL-related, 70% stained positive for mucin 6 [79], which is also expressed in duct and essentially centro-acinar cells [80]. This suggests a ductal/centro-acinar origin for pancreatic serous cysts [79].

### Cilia loss in pancreatic cells *in vivo*

A study with conditionally inactivated *Kif3a* in pancreatic tissue in mice suggests that pancreatic cysts originate from duct cells, as a result of cilia loss [66]. *Pdx1-Cre*^*early*^*;Kif3a f/f* mice were sacrificed at different time points: at two days postnatal, loss of acini was observed and enhancement of lumen between acinar cells and interstitial cells in acini; at 15 days progressive lumen between acini and duct dilation were found. These pathologies all progressed with age and led to acinar tissue being replaced by adipose tissue, severe fibroses, fluid-filled cysts and extensive ductal dilation (age 6 to 12 months). Cilia were lacking in all pancreatic cells. To further identify the cells of origin, conditional knock-out mice were developed for *Kif3a* inactivation in islet cells only, as well as in islet and acinar cells. In mice lacking *Kif3a* in islet cells, no morphological abnormalities were observed. Cilia were only present in duct and reduced in islet cells, suggesting that pancreatic cysts originate from ductal cells [66].

## Conclusions

We argue that VHL disease can serve as an excellent model to improve the understanding of pathophysiology of pancreatic cysts in general. *In vitro* studies support a role for pVHL in microtubule stabilization and subsequent cilia maintenance. Loss of cilia is directly related to renal cyst development in VHL and in other renal cystic syndromes [51]. Other cell aspects are also involved; pVHL influences assembly of the extracellular matrix as well as the cytoskeleton, including cell polarity.

In *Pdx1-Cre*;*Vhlh f/f* mice with pancreatic-specific loss of *Vhlh*, pancreatic cysts were observed after 16 to 18 months [71]. Conditional *Kif3a* knock-out in pancreatic duct cells in mice also resulted in cysts, but changes were already observed after 2 days [66]. Assuming that cysts mainly result from cilia loss, these data suggest that loss of additional alleles might be necessary for *Vhlh*-driven pancreatic cyst development. In contrast, exome sequencing of sporadic human pancreatic serous cysts only found chromosome 3p loss/*VHL* mutations as recurrent genetic lesions, suggesting that *VHL* loss is sufficient for pancreatic cyst development. The high prevalence of pancreatic cysts in patients with VHL disease supports this notion. Differences in findings might be attributed to differences in species, since in general mice seem to be relatively resistant to *VHL* loss when compared to humans [81], indicating that in humans *VHL* loss alone might be sufficient for pancreatic serous cyst development.

Monogenetic diseases like VHL provide insights which can be translated to pancreatic cysts in general. Better understanding and identification of genes regarding pancreatic cyst development will probably provide new directions for diagnostics, follow-up and treatment options. Future studies should focus more on *VHL* and also other E3 ubiquitin ligase genes, which appear to be involved in pancreatic cysts.

## Abbreviations

aPKC: Atypical protein kinase C; ECM: Extracellular matrix; HIF: Hypoxia inducible factor; KAP: Kinesin-associated protein; LOH: Loss of heterozygosity; PAR: Partitioning defective proteins; Pdx1: Pancreatic and duodenal homeobox 1; pNET: Pancreatic neuroendocrine tumor; pVHL: von Hippel-Lindau protein; RCC: Renal cell carcinoma; VEGF: Vascular endothelial growth factor; VHL: von Hippel-Lindau.

## Competing interests

The authors declare that they have no competing interests.

## Authors’ contribution

SA: planning/conducting the review, collecting and analysis/interpretation of literature, drafting the manuscript. EV: analysis/interpretation of literature, critical review and revision of the manuscript. HD: critical review and revision of the manuscript. AB: critical review and revision of the manuscript. AW: critical review and revision of the manuscript. RG: analysis/interpretation of literature, critical review and revision of the manuscript. TL: analysis/interpretation of literature, critical review and revision of the manuscript. All authors read and approved the final manuscript.
